# Ginger (Zingiber officinale) induces apoptosis in Trichomonas vaginalis in vitro 

**Published:** 2016-11

**Authors:** Mohsen Arbabi, Mahdi Delavari, Zohre Fakhrieh Kashan, Mohsen Taghizadeh, Hossein Hooshyar

**Affiliations:** 1 *Department of Medical Parasitology, School of Medicine, Kashan University of Medical Sciences, Kashan, Iran. *; 2 *Department of Medical Nutrition, School of Medicine, Kashan University of Medical Sciences, Kashan, Iran.*

**Keywords:** *Trichomonas vaginalis*, *Ginger*, *Zingiber officinale*, *Extract*, *Apoptosis*, *3-(4-5-dimethylthiazol-2-yl)-2*, *5-diphenyltetrazolium bromide assay*, *IC*_*50*_, *In vitro*

## Abstract

**Background:**

Trichomoniasis is the most common sexually transmitted protozoan diseases in the worldwide. Metronidazole is the choice drug for trichomoniasis treatment, however, metronidazole resistant *Trichomonas vaginalis* (*T.vaginalis*) has been reported. Natural products are the source of most new drugs, and *Zingiber officinale* (Ginger*)* is widely used ingredient in the traditional medicine.

**Objective:**

The aim of the present study was to determine the effect of different concentrations of the ginger ethanol extract on the growth of *T.vaginalis* trophozoites in vitro.

**Materials and Methods:**

In this experimental study, 970 women who were attend in Kashan health centers were examined for *T. vaginalis*. Of them, 23 samples were infected with *T.vaginalis*. Three *T. vaginalis* isolates were cultured in a TYI-S-33 medium. The effect of ginger ethanol extracts and its toxicity in different concentrations (25, 50, 100, 200, 400, 800 µg/ml) on mouse macrophages were measured in triplicate exam by MTT [3-(4,5-dimethylthiazol-2-yl)-2,5-diphenyltetrazolium bromide] assay. The effect of ginger on apoptosis induction was determined by Flow cytometry.

**Results:**

The IC_50_ of ginger and metronidazole were 93.8 and 0.0326 µg/ml, respectively. 12, 24 and 48 hr after adding different concentrations of extract on mouse macrophages, fatality rates in maximum dose (800 µg/ml) were 0.19, 0.26 and 0.31 respectively. Flow cytometry results showed the apoptosis rate following treatment with different concentrations of the extract after 48 hr were 17, 28.5, 42.1, 58.8, 76.3 and 100% respectively, while in the control group was 2.9%.

**Conclusion:**

Ginger ethanol extract induces programmed death in *T. vaginalis*. It is recommended that due to the known teratogenic effect of metronidazole, ginger can be considered as an alternative drug for metronidazole.

## Introduction

Trichomoniasis is a common pathogenic sexually transmitted diseases (STDs) caused by the motile parasitic protozoan called *Trichomonas vaginalis* (*T. vaginalis*). The World Health Organization (WHO) has estimated that there are 248 million new cases *T. vaginalis* each year occur in men and women and nearly 90% of these infections occurred among people living in resource-limited settings. This parasite can live in the urogenital tract and infect any sexually active person, especially in people with multiple sexual partners and other venereal diseases (1-3). 

The cumulative information suggests that* T. vaginalis* has an important role in increasing getting or spreading other sexually transmitted infections.* T. vaginalis* is an important cofactor in promoting the spread of human immunodeficiency virus (HIV), and, may have a major impact on the epidemic dynamics of HIV in some countries (4-9). *Trichomoniasis* causes a wide range of symptoms, from a relatively asymptomatic condition to mild irritation and severe inflammation. In men, the infection usually happens in the urethra, burning after urination or ejaculation, or some discharge from the penis. Among women, the clinical features include vaginal discharge, discomfort with urination, itching, redness or soreness of the genitals, and abdominal pain. Without treatment, the infection can last for months or even years (4, 10, 11). 

Recent evidence also suggested a link between *T. vaginalis* infection in males and later aggressive prostate cancer. Also in women a relationship between infection and cervical cancer, atypical pelvic inflammatory disease and infertility have been reported (12, 13). In long-term trichomoniasis, the parasite can colonize and persist within the vagina. In this process, lymphocytes, erythrocytes and interactions with vaginal epithelial cells are all thought to play important roles. *T. vaginalis* can also bind to host extracellular matrix proteins. Experimental evidence showed that adhesion of parasite of the vaginal epithelial cells take place through specific protein- protein interactions. Adhesion of the parasite is a critical step in their virulence and pathogenesis (8, 12). 

The nitroimidazoles are the only class of medications known to be effective against T. vaginalis infections. Of these drugs, metronidazole and tinidazole have been cleared and approved drugs by the Food and Drug Administration (FDA) (14). Although metronidazole has been the mainstay of treatment for several decades and the cure rate with this drug is high, the long term application of the drug has caused the development of drug-resistant to *T. vaginalis*, and is the main reason of treatment failure. In addition, it was reported that metronidazole had side effects and induces hypersensitivity reactions, nausea, dizziness and manifesting as dermatological symptoms, can be occurred (15). 

Therefore, there is necessary to develop new effective drugs to treatment trichomoniasis. Some medicinal plants are investigated for this proposes (16). The aromatic rhizome of *Zingiber officinale* is the source of ginger, a spice used for centuries to add flavour in cooking and juices among these promising medicinal agents (17). For centuries, ginger has been used in traditional medicine for stroke and heart disease, indigestion and nausea, malabsorption, bacterial and fungal infections, ulcers, cancer, diabetes and respiratory disorders.

Based on different studies, ginger contains flavonoids, alkaloids, coumarins, saponins, tannin, alkaloid and glycosides and have anti-bacterial, anti-parasitic and antioxidant effects (17-20). Pharmacological components of ginger, including essential oils, zingiberol, zingiberone, zingiberene, and pungent agents such as gingerol and with other gingerol analogues such as the shogaol, paradol and zingerone have been identified (20). Phytochemical studies showed that the major components of ginger are shogaols paradol, zingerone and gingerols. These agents have the ability to control the transformative processes of hyperproliferative, inflammation and carcinogenesis.

The phenol compounds derived from ginger (gingerol and shogaol) have many interesting physiological and pharmacological activities. Although, it has been used for centuries, this plant still attracts extensive research attention (21). It is undoubtedly necessary the research for new therapeutic targets, which is essential to the rational development of new anti-*T.vaginalis* compounds structurally distinct from 5-nitroimidazoles (22, 23). 

Therefore, the study of plants used by indigenous medicine is important to interconnect traditional medicine and biotic environment preserving the indigenous ancient knowledge. Considering the indigenous ethnopharmacology as a contributor to the systematic screening of plants with antiprotozoal activity, and the need for new, safe and effective drugs for trichomoniasis treatment, the current study was designed to address the possible antiparasitic effect of ginger extract against *T. vaginalis* infection under in vitro condition.

## Materials and methods

**Preparation of plant extracts**

The fresh ginger plant was prepared from market and approved by nutrition department of Kashan University of Medical Sciences. It was decoction at 60^o^C for 60 min [1 : 10; (w : v)]. Alcoholic extracts were freeze-dried, and the work solution was prepared at 8.0  mg/ml in ultrapure water, sterilized by filtration (0.22  µm) and stored at -20^o^C. 

**Parasite culture**

In this experimental study, 970 women were referred to Kashan Medical Centers from October 2013 to August 2014 were examined for *T. vaginalis*. From all participants vaginal discharge samples were taken. Of them, 23 samples were infected by *T. vaginalis*. Three isolates of *T. vaginalis* were cultured in a TYI-S-33 medium and the effect of the ginger extract was studied. Kashan University of Medical Sciences Ethics committee approved the study protocol and consent form was taken from all participants. 

The diagnosis was based on clinical and microscopic visualization (Olympus optical microscope model BX40) of motile *T. vaginalis* parasites on a prepared slide from vaginal discharged. The parasites were axenically grown in standard TYI-S33 (trypticase-yeast extract-maltose) medium (pH=6.8) supplemented with 10% Fetal Calf Serum (FCS), vitamin mixture and 100 U/ml penicillin and streptomycin mixture at 37^o^C. The culture routinely grasped a concentration of 5×10^5^ cells/ml in 48 hr. 

**Anti-trichomonal assay**

Reproducible surveillance of the drug susceptibility status of the anaerobic protozoa *T.vaginalis* was tested as earlier described (24). A density of 5×10^5^, trophozoites /ml were incubated in the presence of serially diluted metronidazole (0.025-4 µg/ml) and alcoholic extract of the ginger (25-800 µg/ml) in the TYI-S33 culture media in 48-well plates at 37^o^C as triplicate. Metronidazole and TYI-S33 culture media without any drug were used as positive and negative controls, respectively. Cells were checked for viability at different time intervals between 12-48 hr. The number of parasites in each well plate was counted after12, 24 and 48 hr by trypan blue staining. After counting the number of parasites, half maximal inhibitory concentration (IC_50_) was determined by Graph Pad prism5 software(GraphPad Software, Inc.USA) and growth inhibition percentage was calculated using the following formula: (A-B)/A×100

A=Average number of trophozoites in control group, B=Average number of trophozoites in test group 

**Toxicity evaluation of ginger extract**

The different concentrations) 25, 50, 100, 200, 400, 800 µg/ml) of gingers extract’s cytotoxic effect on mouse macrophage cells was evaluated at three different times by using MTT [3-(4,5-dimethylthiazol-2-yl) -2,5-diphenyltetrazolium bromide (25). The peritoneum macrophages were isolated from the peritoneum of BALB/c mice by injection the cold phosphate-buffered saline (PBS) and re-aspiration. 10^5^ cells/well macrophages were seeded on each well of the 96-well plates and supplemented with 10% FCS at 37^o^C under 5% CO_2_ and treated with different concentrations of the ginger extract for 24 hr. Cells treated with no extract were taken as control groups. 20 µl MTT reagents (5 mg/ml, pH 7.4) in fresh culture medium was added to each well after 48 hr, then the plates were incubated for 3-5 hr at 37^o^C under 5% CO_2_. After that, the supernatant was removed from wells and 100 µl of Dimethyl sulfoxide (DMSO) was added to each well.

Since 15 min, optical density (OD) of each well was read at 570 nm by an ELISA reader. Percentage of killing macrophages was determined based on the optical absorbance in test and control groups using the following formula (18): killed macrophage (%)=1-(AT-AB)/(AC-AB)×100. 

AB is the OD of the blank well, AC is the OD of the untreated samples and AT is the OD of treated samples.

**Flow** **cytometry analysis of cell death**

The Annexin-V FLUOS Staining Kit (Bio-vision, USA) was used for the detection of apoptotic and necrotic cells according to the manufacturer's protocol. Briefly, trophozoites was cultured in 24-well plates (5×10^6^ parasites/well) in the absence (negative control group) and the presence of 25, 50, 100, 200, 400 and 800 μg/ml of ginger and were incubated at 24^o^C. According to the flow cytometry kit instruction, the trohozoites were collected after 48 hr incubation and centrifuged at 2000 RPM for 5 min, then supernatant was discharged, and 500μl binding buffer, 5 μl Annexin-V and 5μl Propidium iodide (PI) were added to the residue. The samples were incubated at room temperature and dark condition for 5 min. Absorbed color intensity in cells was observed by flow cytometry (2005 by Partec Gmbh Munster, Germany). The results were analyzed by FlowJo software, and the level of apoptosis was determined. 

**Statistical analysis**

All experiments were performed at least three times. All data were recorded in a Microsoft excel worksheet and analyzed using the SPSS software package (Ver. 16). Results are expressed as mean±SD. The significant differences among values were analyzed using analysis of Student’s *t* test and variance (one-way ANOVA) coupled with post-hoc least significance difference (LSD). P<0.05 is considered significant.

## Results

**G** **rowth inhibition**

The number of viable *T.vaginalis *trophozoites was decreased compared to the control group with an exposure to ginger alcoholic extract after 12 hr and 24 hr. The alive throphozoites were decreased significantly by increasing concentration of extract and culture time. There were no motile and alive trophozoites at 800 µg/ml concentration of the extract after 48 hr (Table I). The number of protozoa fell to zero in a concentration of 800 µg/ml after 48 hr. The IC_50_ of ginger and metronidazole against* T.vaginalis* after 24 hr was calculated 93.8 µg/ml and 0.0326 µg/ml respectively. 

**MTT assay**

Percentage of killing macrophage cells following exposure to the different concentrations of the ginger alcoholic extract is shown in table II. After 12 hr ginger alcoholic extract in 25-800 µg/ml concentrations the fatality rate (%) was observed between 0.11-0.19, it was between 0.15-0.26 and 0.18-0.31 after 24 and 48 hr respectively. Toxicity of the ginger alcoholic extract at the highest concentration (800 μg/ml) and time (48 hr) was 0.31% in macrophage cells.

**Flow cytometric results**

**Figure 1:**
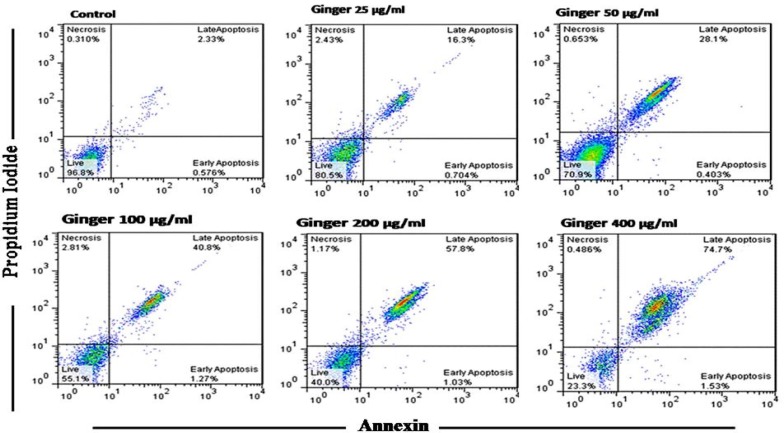
Flow cytometry results showed the percentage of apoptotic, live and necrotic cells in treating *T.vaginalis* trophozoites with ginger alcoholic extract after 48 hr in different concentrations

Flow cytometry results showed induced apoptosis (early and late apoptosis) following treatment trophozoeits with different concentrations of ginger ethanol extract. Early apoptosis in 25, 50, 100, 200 and 400 µg/ml were 0.70%, 0.40%, 1.27%, 1.03% and 1.53%, respectively, and also for late apoptosis these percentages were 16.3%, 28.1%, 40.8%, 67.8% and 74.7%, respectively (Figure 1).

**Table I:** Comparison of different concentrations of Ginger ethanol extract of *T. vaginalis*, after 12 hr, 24 hr and 48 hr exposure

**Ginger** ** concentrations** ** (g/ml** **µ)**	***T. vaginalis*** ** trophozoite (× 10** ^4^ **)**		
	**48 hr**	**24 hr**	**12 hr**
25	1.76 ± 34.7	2.65 ± 37	3.51 ± 45.7
50	5 ± 25	4.73 ± 29.6	3.21 ± 36.3
100	3.5 ± 18.7	3.46 ± 24	5 ± 25
200	2 ± 10	2.52 ± 17.7	5.3 ± 20.3
400	2 ± 6	4.51 ± 9.33	2.51 ± 16.3
800	0	1.53 ± 4.7	4.51 ± 10.33
Positive control (0.1μg/ml)	-	-	1.5 ± 8.3
Negative control	6.5 ± 88.3	51.3 ± 53.66	2.51 ± 38.33

Data presented as mean±SD. (Statistical comparison of groups, p=0.001)

One-Way ANOVA test

**Table II:** Comparison of the fatality rate (%) ginger ethanol extract on macrophages isolated from mice, 12 hr, 24 hr and 48 hr after exposure

**Ginger ** **concentrations (** **g/ml** **µ)**	**Killed macrophages (%)**		
	**12 hr**	**24 hr**	**48 hr**
25	0.11	0.15	0.18
50	0.12	0.16	0.20
100	0.12	0.20	0.23
200	0.13	0.21	0.24
400	0.14	0.22	0.26
800	0.19	0.26	0.31

One-Way ANOVA test.

Statistical comparison of groups, p=0.001

## Discussion

Trichomoniasis is one of the most common sexually transmitted diseases in humans. individuals with this chronic infection are at high risk for HIV seroconversion (4). However, increasing resistance to drugs such as metronidazole poses a serious problem, and new effective tactics are needed to prevent this infection. The current medications and the first line drugs for treatment of *T.vaginalis* are metronidazole and tinidazole, both 5-nitroimidazole drugs, are approved by the FDA for this infection. However, using metronidazole is limited because of their high toxicity, high doses and development of drug resistance (14, 15, 26).

The present study demonstrated that administration of the ginger extract to in vitro condition has potential anti-Trichomonas activity at a concentration 800 µg/ml on the Iranian strain of *T.vaginalis*. These findings are in the same line with a previous study that attributed the significant effect on parasite following administration of ginger to the disturbance in parasite due to the anti-parasitic activity of gingerol, shogaol, and hexahydro-curcumin, a constituent isolated from the roots of ginger which may have a direct effect on the vitality and viability of parasite (17, 20, 21). Trichomonad cytopathogenicity is a multifactorial process involving events such as cytoadherence and immune evasion. Cytoadherence is a key property for colonization and infection by *T.vaginalis *and is mediated principally by two groups of molecules named adhesions and cysteine proteinases (CPs), which are expressed on the parasite surface. Besides, iron is an important nutrient for *T. vaginalis*, which it cannot be synthesized on its own and must receive it by lysis of erythrocytes (27). It was shown that ginger has components such as Saponin (28). 

Saponins are detergents, and there is a possibility that detergent action may affect the membrane bi-lipid layer and lead to a decrease in the cytoadherence. Saponins also inhibit proteolytic activity of parasite’s CPs that are important for adherence, nutrition acquisition and virulence of the parasites. Adhesion protein AP65 is an important adhesion targeted to both surface membrane and hydrogenosome. It is centrally involved in cytoadherence (24, 29). According to Tiwari and coworkers, saponins prevent the suppression of pro-inflammatory cytokines induced by *T. vaginalis*, suggesting one of the possible mechanisms of saponins against Trichomonas action (30).

Previous studies have also shown that treatment of *T. vaginalis *with pro-apoptotic drugs and metronidazole leads to a form of non-necrotic cell death with some features resembling apoptosis (16, 31). The use of caspase inhibitors that abolish the apoptotic process in *T. vaginalis *strongly suggests the presence of caspase-like proteinases in this microorganism (30, 31). Induction of apoptosis or apoptosis-like cell death is one of advantages of ginger against other currently used drugs, including metronidazole, as it does not lead to provocation of an acute immune response. So far, apoptosis has been described in at least nine species of unicellular organisms, including Leishmania (32, 33). 

According to the flow cytometry results in the present study, in the control group, 96.8% of cell were alive and percentages of necrosis, early apoptosis and late apoptosis were 0.310%, 0.576% and 2.33% respectively. It was noted the alive trophozoites in a concentration of 25, 50, 100, 200, 400 µg/ml, were 80.5%, 70.9%, 55.1%, 40%, an 23.1% respectively. In our study, MTT assay was used to determine the IC_50_ rate of ginger extract on the Iranian strain’s *T. vaginalis *trophozoites’s. We have determined an IC_50_ of 93.8μM for gringer and 0.0326μM for metronidazole, which is higher than reported for other plant species )^16^). The reported IC_50_ for extract in this study and metronidazole as control test are similar investigation. Cedillo-Rivera and collegeas found IC_50_ of 0.034 μM for metronidazole that has little difference with our study (34). 

In another study, Azadbakht *et al* showed positive effects of hydro alcoholic extracts of Thymus vulgaris and Myrtus communis against *T. vaginalis*, and reported the IC_50_:0.12 μM for Thymus vulgaris and *IC*_50_:0.34 μM for Myrtus communis, which had high effect in comparison with Grion alcoholic extract that we used in the present study (35).

## Conclusion

In conclusion, our results revealed that the ginger extract can induce apoptosis in *T.vaginalis *by dose- and time-depended manner in in vitro condition. 

More comprehensive studies are needed to survey antitrichomonas activity of the ginger extract in in vitro and in vivo and conditions.

## Note

***This article extracted from M.Sc. thesis. (Zohre Fakhrie Kashan)***
